# A Chair-Based Unconstrained/Nonintrusive Cuffless Blood Pressure Monitoring System Using a Two-Channel Ballistocardiogram

**DOI:** 10.3390/s19030595

**Published:** 2019-01-31

**Authors:** Kwang Jin Lee, Jongryun Roh, Dongrae Cho, Joonho Hyeong, Sayup Kim

**Affiliations:** 1 Deepmedi Research Institute of Technology, Deepmedi Inc., Seoul 06232, Korea; kjlee@deep-medi.com (K.J.L.); dongrae30@deep-medi.com (D.C.); 2 Human Convergence Technology Group, Korea Institute of Industrial Technology (KITECH), 143 Hanggaulro, Ansan 15588, Korea; ssaccn@kitech.re.kr (J.R.); freegore@kitech.re.kr (J.H.)

**Keywords:** cuffless blood pressure monitoring system, hypertension, photoplethysmogram

## Abstract

Hypertension is a well-known chronic disease that causes complications such as cardiovascular diseases or stroke, and thus needs to be continuously managed by using a simple system for measuring blood pressure. The existing method for measuring blood pressure uses a wrapping cuff, which makes measuring difficult for patients. To address this problem, cuffless blood pressure measurement methods that detect the peak pressure via signals measured using photoplethysmogram (PPG) and electrocardiogram (ECG) sensors and use it to calculate the pulse transit time (PTT) or pulse wave velocity (PWV) have been studied. However, a drawback of these methods is that a user must be able to recognize and establish contact with the sensor. Furthermore, the peak of the PPG or ECG cannot be detected if the signal quality drops, leading to a decrease in accuracy. In this study, a chair-type system that can monitor blood pressure using polyvinylidene fluoride (PVDF) films in a nonintrusive manner to users was developed. The proposed method also uses instantaneous phase difference (IPD) instead of PTT as the feature value for estimating blood pressure. Experiments were conducted using a blood pressure estimation model created via an artificial neural network (ANN), which showed that IPD could estimate more accurate readings of blood pressure compared to PTT, thus demonstrating the possibility of a nonintrusive blood pressure monitoring system.

## 1. Introduction

Hypertension is a well-known chronic disease, which affects approximately 25% of all adults in the world according to the World Health Organization (WHO) [1]. Unfortunately, most patients do not know they have hypertension because they do not experience any symptoms until they suffer a cardiovascular disease or stroke, making hypertension a silent killer. Campaigns to measure blood pressure are conducted worldwide to alert people about the importance of its management [2].

Since hypertension is a fatal disease that can result in death or disabilities due to cardiovascular diseases or stroke, it requires an accurate diagnosis and response. A system that can continuously measure blood pressure to alert patients to the dangers of hypertension is required. However, the existing method to measure blood pressure uses a cuff wrapped around the arm, making it cumbersome, and hence, most patients do not measure their blood pressure regularly. Recently, cuffless blood pressure measurement techniques have been continuously researched to improve the convenience of measurement [3,4,5]. The most popular methods for cuffless blood pressure measurement involve the use of pulse transit time (PTT), which is calculated using electrocardiogram (ECG) and photoplethysmogram (PPG) signals.

The PTT has a significantly high correlation with blood pressure [5], and regression analysis methods for measuring blood pressure using PTT have been researched [6,7,8]. However, these methods failed to show sufficient accuracy to be applied in medical devices. Hence, estimation methods using machine learning and deep learning algorithms have been evaluated to improve the accuracy of blood pressure estimation [9,10].

However, while estimating blood pressure using PTT, users must consciously make measurements using ECG and PPG sensors to obtain their blood pressure. To address this issue, techniques for non-intrusive measuring of biometric signals have been developed, thus improving the convenience of measurement [11,12,13]. One of the biometric signals used is the ballistocardiogram (BCG) signal, which measures the movement of the body during cardiac contraction and relaxation. Studies have been actively carried out using radar and polyvinylidene fluoride resin (PVDF) films to measure blood pressure using BCG signals. Further, other studies have estimated blood pressure by calculating PTT using BCG, PPG, or ECG signals for users seated on a chair [14,15]. However, measuring blood pressure using PPG cannot be considered as a fully nonintrusive/unrestricted system because the users must be conscious while measuring their blood pressure, which is inconvenient. This is because users or patients must attach their finger to PPG sensors in order to enable the measurement of PPG signals. Therefore, they would be consciously rigid while having their blood pressure measured via PPG. Moreover, BCG signals are combined with breathing or motion noises, which make it difficult to capture the maximum peak of BCG, resulting in an error in the calculation of PTT using BCG and ECG signals.

To solve this problem, this study developed a fully nonintrusive/unrestricted chair-type cuffless blood pressure monitoring system that utilizes two PVDF films. After measuring two BCG signals from the two PVDF films, the phase difference between the two signals was calculated using the Hilbert transform. Blood pressure was estimated via an artificial neural network (ANN) using this phase difference as a feature value. The applicability of the developed system was verified by comparing the estimated blood pressure with the results from the existing PTT calculation method.

## 2. Materials and Methods

### 2.1. System Summary

**Figure 1:**
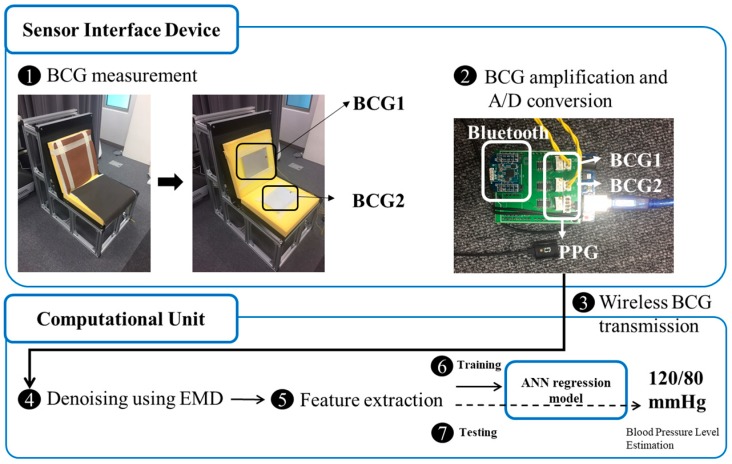
Conceptual diagram of the chair-type unrestricted/nonintrusive blood pressure measurement system. The entire system consists of a sensor interface device and a computational unit. Ballistocardiograms (BCGs) are measured through the polyvinylidene fluoride (PVDF) films from the chair’s back and seat plates and are sent to the computational unit (as indicated by a fine line), which then estimates the blood pressure by extracting the features from the two BCG signals.

In this study, a sofa-type experimental apparatus was fabricated to ensure the measurement of a stable biometric signal. The weight was supported by a steel plate on an aluminum frame, and PVDF films were attached to a urethane foam back and a cushion on the seat, which were then covered with natural leather to create a structure similar to a sofa. The BCG signals were measured in real time using the PVDF films attached to the experimental apparatus. To confirm that the BCG signals were measured accurately, the PPG signals were simultaneously measured as a reference by using a PPG sensor (RP520, Laxtha). The Atmega256 chip (Atmel Corporation, San Jose, CA, USA) was used as a microcontroller and the sampling frequency was set at 100 Hz. The raw BCG signals were sent to a computer system (Core i7, Window 10) via Bluetooth (Parani ESD-200, Sena technologies, Seoul, Korea). The software developed in this study provides features such as noise processing extraction, as well as modeling and functions of blood pressure estimation. The software was developed on a MATLAB base. After the training stage, it can estimate blood pressure at intervals of 10 s through the signals measured via the chair. The concept of the system is illustrated in Figure 1.

### 2.2. Experimental Procedure

This study conducted experiments on 30 adults aged 20–50 years (14 men, 16 women) with wide ranging attributes (35.3 ± 12.5 years, height: 166.1 ± 9.4 cm, weight: 63.3 ± 12.8 kg). The subjects were selected from a group of people with no hypertension. The study was approved by the Public Institution Bioethics Committee designated by the Ministry of Health and Welfare of South Korea (IRB P01-201812-12-001).

In the experiments, the stable blood pressures of the subjects were measured simultaneously using the experimental sofa and a cuff-type blood pressure monitor (HEM-7121, Omron, Kyoto, Japan) after the subjects were given a sufficient rest. The blood pressure was measured five times at intervals of 1 min.

### 2.3. BCG Signal Processing Using Empirical Mode Decomposition

**Figure 2:**
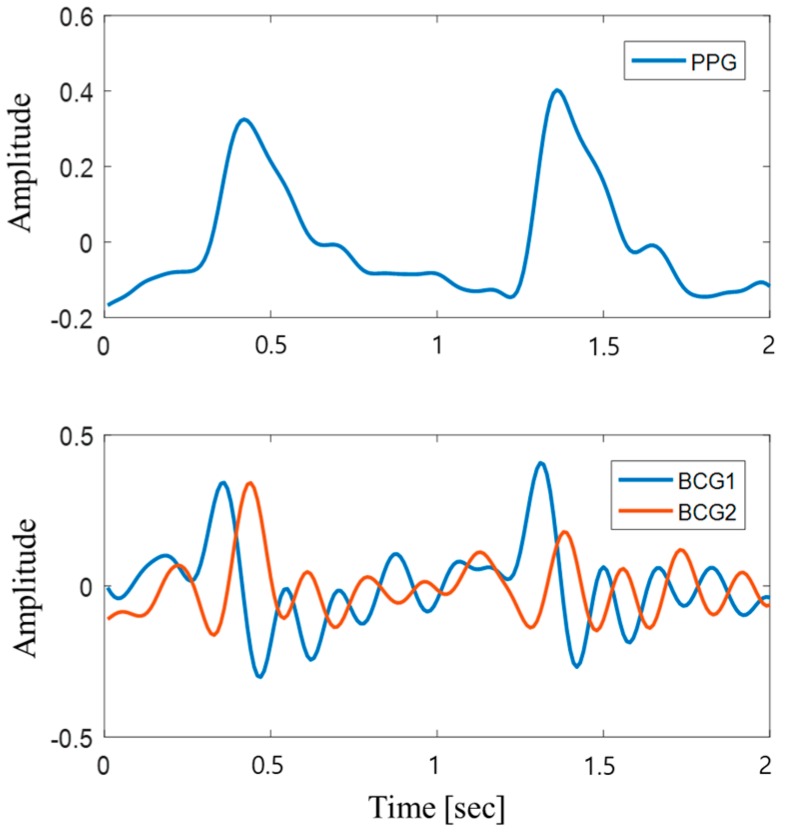
Photoplethysmogram (PPG) signals measured using the developed system as reference signals, which indicate whether the peaks of the BCG are working properly. BCG1 and BCG2 refer to the signals measured from the back and seat plates, respectively.

**Figure 3:**
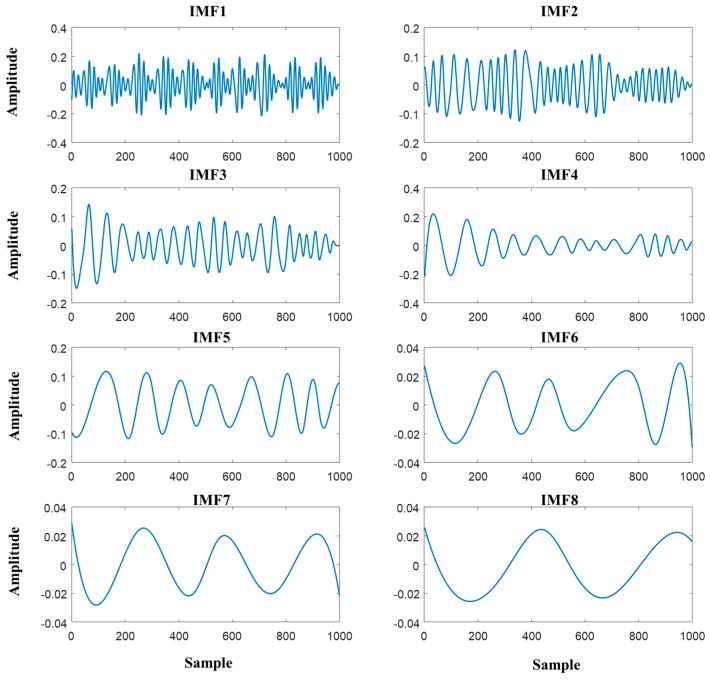
BCG1 signals decomposed through empirical mode decomposition (EMD), out of which IMF1 (intrinsic mode function) was used.

BCG signals consist of various other signals such as breathing signals and motion noises, particularly many motion noises that occur at low frequencies. Since heartbeat signals can be measured in the bandwidth of 0.5–6 Hz, BCG signals without motion noises were obtained in this study by applying a third-order Butterworth band-pass filter with cut-off frequencies of 0.5–6 Hz. Figure 2 shows the comparison of PPG signals with the filtered BCG signals from the back and seat plates. Although the signals underwent preprocessing through a band-pass filter, it is difficult to effectively remove the cardiorespiratory signals from the BCG signals. Empirical mode decomposition (EMD) was used to remove the noise signals because EMD is known to be a better method when compared to wavelet decomposition [16]. As one of the decomposition methods proposed by Huang et al. [17], EMD decomposes the signals via intrinsic mode functions (IMFs) depending on the level of local frequencies. The decomposed signals are shown in Figure 3. Only the first IMF signal was used in this study. The EMD method performs analysis according to the following procedure:

(1) Identify the local maxima and local minima of the given time-series signals.
(2) Use interpolation to estimate the upper and lower envelopes by connecting the local maxima and minima values, respectively.
(3) Calculate the mean envelope by averaging the upper and lower envelopes determined in the above point.
(4) Extract new time-series signals by subtracting the mean envelope determined in the above point from the original signals. These extracted signals are defined as IMF.
(5) Set the time-series signals from which the extracted IMF is removed as new original signals and repeat steps (1) to (4). Define the new IMF until the newly designated original signals are expressed as a monotone function or have only one extreme value and no more new time-series signals can be extracted.

### 2.4. BCG Data Analysis and Feature Extraction

**Figure 4:**
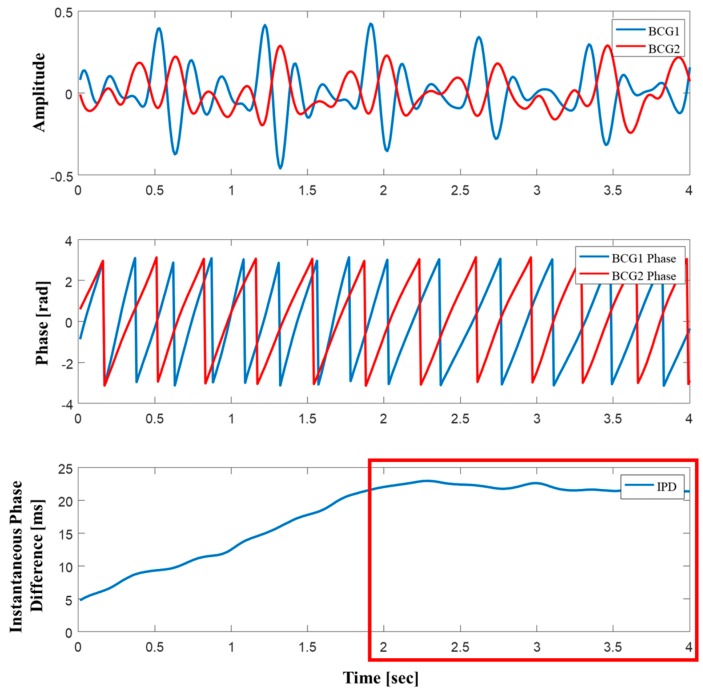
Process to calculate instantaneous phase difference (IPD).

The estimation of blood pressure using PTT is greatly affected by the accuracy of finding the peak i.e., the blood pressure cannot be estimated correctly if the peak is missed from signals. Hence, while the peak was not sought, the same features as PTT were extracted from the BCG signals by using the instantaneous phase difference (IPD) method. Many researchers have used this method to estimate blood pressure from PPG signals, demonstrating its significant correlation with PTT [18,19]. The instantaneous phase was obtained from the first IMF of the two BCG signals using Hilbert transform, and then deducted to determine the IPD (see Figure 4). As shown in Figure 4, we extracted features from the part between the two BCG signals with less phase difference. To determine the PTT for comparison with IPD, the peak was found by using adaptive threshold detection [20].

### 2.5. ANN

To create a blood pressure estimation model, a regression analysis model was created using an ANN. An ANN is a mathematical model consisting of numerous processing elements with a hierarchical structure in which the relationships between inputs and outputs are studied by adjusting the weighted values repeatedly across previous input data and the corresponding output data. It can be used to estimate a very complex nonlinear function. In fact, a multilayer feed-forward back propagation ANN with one hidden layer is sufficient for fitting a continuous function. The median of the IPD value of two BCG signals and personal information (height, weight, age) was entered in the ANN input layer as the feature values, and 25 neurons of the hidden layer were used. The SBP and DBP values were estimated from the two output neurons in the output layer. The ANN was trained using the Levenberg–Marquardt algorithm with 70% of the sample data as training set, 15% as validation set, and the remaining 15% as testing set. The model was then trained and evaluated via 10-fold cross validation to obtain the optimum model.

## 3. Results

### 3.1. IPD

**Figure 5:**
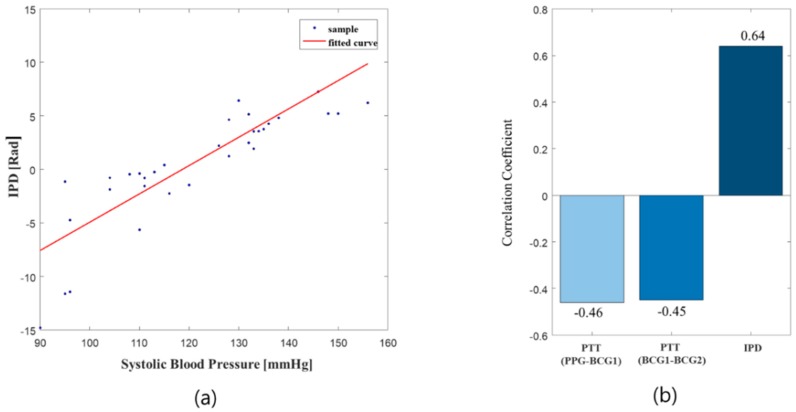
Relationships between systolic blood pressure and PTT/IPD, (**a**) systolic blood pressure and IPD, (**b**) correlation coefficient of systolic blood pressure with PTT/IPD.

To examine the relationship between IPD and blood pressure, the relationship between the median of the IPD and the systolic blood pressure was observed [19]. As shown in Figure 5, the IPD was correlated with the blood pressure i.e., the higher the blood pressure, the greater the difference of IPD. The correlation coefficient between PTT and the systolic blood pressure also suggests that the blood pressure correlates more with IPD compared to PTT.

### 3.2. BP Estimation Model

**Figure 6:**
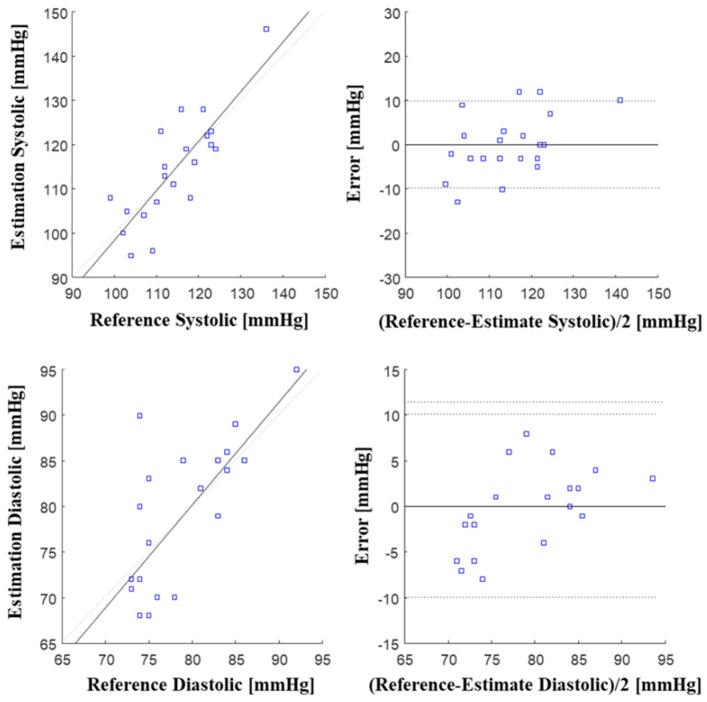
Bland–Altman plot and regression plot in systolic and diastolic periods.

Table 1 shows the mean error (ME) and standard deviation (STD) calculated using the results of the BP estimation model created using ANN and the BP results measured with a commercial cuff-based blood pressure monitor. In this table, the ME and STD values are evaluated according to the American National Standards Institute/Association for the Advancement of Medical Instrumentation/International Organization for Standardization (ANSI/AAMI/ISO) 2013 protocol (ME < 5 mmHg, STD < 8 mmHg) [21]. As shown in Table 1, the mean deviations of the systolic and diastolic blood pressures are 0.01 mmHg and 0.05 mmHg respectively, and the standard deviations are 6.7 mmHg and 5.8 mmHg respectively, which are within the recommended criteria. These values were also within the recommended criteria when the BP estimation model was created with PTT as input values. However, the BP estimation model that used IPD as input values showed a higher accuracy for the systolic blood pressure, whereas for the diastolic pressure, the model created using PTT obtained from two BCGs showed a higher accuracy. Figure 6 shows the Bland-Altman plot and the regression plot of the estimation model using IPD, which display high accuracy.

## 4. Discussion and Conclusions

In this study, a chair-type system that can measure blood pressure in a nonintrusive manner using PVDF films and two BCG sensors was developed. While chair-type blood pressure monitoring systems have been developed in the past [14,15], most of them had low utility because they require users to be conscious during measurement. However, the system developed in this study can measure blood pressure even if the user simply sits on a chair via BCG sensors installed on the back and seat plates, thus improving convenience. BCG signals have a limitation toward increasing the accuracy of blood pressure estimation because they are mixed with various noises, and hence cannot accurately detect peak positions and often fail to capture the PTT. However, the system proposed in this study could accurately estimate blood pressure (as shown in Table 1) even if it did not capture the location of the peak because it uses IPD, which is highly correlated with blood pressure as shown in Figure 4. The system developed in this study also has certain limitations. The number of test subjects is smaller than the number of subjects (a minimum of 85) recommended for evaluating blood pressure monitors by the AAMI, and the accuracy for detection of hypertension and hypotension is sometimes low. This may likely be due to the small sample size, which leads to the belief that the performance can be improved if more data are collected.

The experimental results of this study showed the possibility of a nonintrusive/unrestricted blood pressure estimation system, which utilizes IPD. This system can continuously monitor blood pressure in a nonintrusive manner while the user is simply sitting on a chair. Since this study only estimated blood pressure in a state of rest, further research is needed in the future to determine whether the proposed system can accurately measure blood pressure for varying situations, such as a blood pressure that has returned to a normal level after having been increased during exercise. It should also be verified for applicability to use for both home and hospital living.

## Figures and Tables

**Table 1:** Mean error (ME) and standard deviation (STD) values of systolic and diastolic blood pressures estimated via pulse transit time (PTT) and instantaneous phase difference (IPD).

	Systolic		Diastolic	
	ME	STD	ME	STD
PTT (PPG-BCG1)	0.9805	7.6471	−0.1467	5.5148
PTT (BCG1-BCG2)	−0.7616	7.5696	0.0341	4.0625
IPD	0.0123	6.7452	0.0532	5.8317

## Abbreviations

The following abbreviations are used in this manuscript:

WHO	World Health Organization
PTT	Pulse Transit Time
ECG	Electrocardiogram
PPG	Photoplethysmogram
BCG	Ballistocardiogram
PVDF	Polyvinylidene fluoride resin
ANN	Artificial Neural Network
EMD	Empirical mode decomposition
IMF	Intrinsic mode function
IPD	Instantaneous phase difference
ME	Mean error
STD	Standard deviation
ANSI	American National Standards Institute
AAMI	Association for the Advancement of Medical Instrumentation
ISO	International Organization for Standardization

## References

[1] World Health Organization (2015). World Health Statistic 2015.

[2] World Health Organization (2014). A Global Brief on Hypertension, Silent Killer, Global Public Health Crisis.

[3] Peter L., Noury N., Cerny M. (2014). A review of methods for non-invasive and continuous blood pressure monitoring: Pulse transit time method is promising. IRBM.

[4] Ahmad S., Chen S., Soueidan K., Batkin I., Bolic M., Dajani H., Groza V. (2012). Electrocardiogram-assisted blood pressure estimation. IEEE Trans. Biomed. Eng..

[5] Mukkamala R., Hahn J.O., Inan O.T., Mestha L.K., Kim C.S., Toreyin H., Kyal S. (2015). Toward ubiquitous blood pressure monitoring via pulse transit time: Theory and practices. IEEE Trans. Biomed. Eng..

[6] Poon C., Zhang Y. Cuff-less and noninvasive measurements of arterial blood pressure by pulse transit time. Proceedings of the 27th Annual International Conference of the IEEE Engineering in Medicine and Biology Society (EMBC).

[7] Kumar N., Agrawal A., Deb S. Cuffless BP measurement using a correlation study of pulse transient time and heart rate. Proceedings of the International Conference on Advances in Computing, Communications and Informatics (ICACCI).

[8] Ding X., Yan B.P., Zhang Y., Liu J., Zhao N., Tsang H.K. (2107). Pulse Transit Time Based Continuous Cuffless Blood Pressure Estimation: A New Extension and A Comprehensive Evaluation. Sci. Rep..

[9] Kachuee M., Kinani M.M., Mohammadzade H., Shabany M. (2017). Cuff-less Blood Pressure Estimation Algorithms for Contentious Health-care Monitoring. IEEE Trans. Biomed. Eng..

[10] Su P., Ding X., Zhang Y., Liu J., Miao F., Zhao N. Long-term blood pressure prediction with deep recurrent neural networks. Proceedings of the IEEE EMBS International Conference on Biomedical & Health Informatics (BHI).

[11] Kranjec J., Beguš S., Drnovšek J., Geršak G. (2014). Novel Methods for Noncontact Heart Rate Measurement: A Feasibility Study. IEEE Trans. Instrum. Meas..

[12] Shu Y., Li C., Wang Z., Mi W., Li Y., Ren T. (2015). A Pressure Sensing System for Heart Rate Monitoring with Polymer-Based Pressure Sensors and an Anti-Interference Post Processing Circuit. Sensors.

[13] Liu M., Jiang F., Jiang H., Ye S., Chen H. (2017). Low-power, noninvasive measurement system for wearable ballistocardiography in sitting and standing positions. Comput. Ind..

[14] Kim C., Carek A.M., Mukkmala R., Inan O.T., Hahn J. (2014). Ballistocardiogram as Proximal Timing Reference for Pulse Transit Time Measurement: Potential for Cuffless Blood Pressure Monitoring. IEEE Trans. Biomed. Eng..

[15] Tang Z., Tamura T., Sekine M., Huang M., Chen W., Yoshida M., Sakatani K., Kobayashi H., Kanaya S. (2017). A Chair-based Unobtrusive Cuffless Blood Pressure Monitoring System Based on Pulse Arrival Time. IEEE J. Biomed. Health Inform..

[16] Sadek I. (2018). Ballistocardiogram Signal Processing: A Literature Review. arXiv.

[17] Huang N.E., Shen Z., Long S.R., Wu M.C., Shih H.H., Zheng Q., Liu H.H. (1998). The empirical mode decomposition and the Hilbert spectrum for nonlinear and non-stationary time series analysis. Proc. R. Soc. Lond. A Math. Phys. Eng. Sci..

[18] Sugita N., Obara K., Yoshizawa M., Abe M., Tanaka A., Homma N. (2015). Techniques for estimating blood pressure variation using video images. Conf. Proc. IEEE Eng. Med. Biol. Soc..

[19] Rapalis A., Janušauskas A., Marozas V., Lukoševičius A. (2017). Estimation of blood pressure variability during orthostatic test using instantaneous photoplethysmogram frequency and pulse arrival time. Biomed. Signal Process. Control.

[20] Shin H.S., Lee C., Lee M. (2009). Adaptive threshold method for the peak detection of photoplethysmographic waveform. Comput. Biol. Med..

[21] Association for the Advancement of the Medical Instrumentation (2002). American National Standard for Electronic or Automated Sphygmomanometers.

